# COVID-19: how can travel medicine benefit from tourism’s focus on people during a pandemic?

**DOI:** 10.1186/s40794-022-00182-6

**Published:** 2022-12-01

**Authors:** Irmgard L. Bauer

**Affiliations:** College of Healthcare Sciences, Academy - Tropical Health and Medicine, James Cook University, Townsville, QLD 4811 Australia

**Keywords:** SARS-CoV-2, Risk Perception, Mass Gatherings, Tourism Employees, Discrimination, Infectious Diseases, Health Literacy, Responsible Tourism, Public Health

## Abstract

In 2020, COVID-19 affected every aspect of life around the globe. The spread of SARS-CoV-2 through travel led to lockdowns, travel bans and border closures, crippling the tourism industry. Without tourists, there would be no tourism industry—and no travel medicine. Therefore, scholars started to research the human aspect of tourism immediately to develop strategies for economic recovery. The resulting insights are useful for travel medicine not only to see how tourism dealt with a medical crisis but also to understand travellers better who may be seeking health advice during and after a pandemic.

This article presents tourism research of 2020 covering risk perception and travel intentions including mass-gatherings, the use of technology to protect from infection, impacts on tourism workers, residents’ reactions to potentially infected travellers, discrimination, and racism. A potential fork in the road to tourism’s future may have implications for travel health practitioners. Research recommendations conclude the paper. Understanding the industry response during the early days of panic and uncertainty may help prepare not only appropriate guidelines for travellers but also clearer instructions for tourism, transportation, and hospitality in anticipation of the next pandemic.

## Introduction

In November 2019, cases of a pneumonia of unknown cause appeared in Wuhan/China, reported to the World Health Organisation (WHO) on 31 December. Early January 2020, the virus SARS-CoV-2 was isolated. On 12 February, the WHO named the resulting disease COVID-19, declaring it a global pandemic on 11 March. From early 2020, publication floodgates opened from many medical specialties (PubMed on 6 July 2021 for ‘COVID-19’ yielded 150,445, six months later 213,484 results). Early health advice for the public, ‘personal non-pharmaceutical protection interventions (PNPIs)’, included cough and sneeze etiquette, self-isolation, avoiding contact/touch, social distancing, hand hygiene, mask wearing, all reasonable textbook instructions many of which were only feasible in affluent countries. From major outbreaks in China and Italy, the virus spread around the world. Travel, a key facilitator of the spread, was first restricted and then prohibited nationally and internationally in many countries via suspension of visa-on-arrival policies, travel restrictions/bans and closed borders. Not always was the relationship between health and politics harmonious or directives aligned.

Media coverage and social media posts during a crisis influence risk perceptions and travel intentions [1, 2]. From the start, WHO alerted to a massive ‘infodemic’, an over-abundance of correct and false messages making it difficult for people to extract useful information, and attempted to debunk myths with accurate information on social media and collaborate with platforms to mitigate the damage [3]. This attempt was unsustainable and unrealistic considering the volume of data and skills necessary to spot the difference [4]. It was difficult to agree on what was reality and what were ‘alternative facts’ or strategic misinformation. COVID-19-misinformation has been deplored in medicine, where it provides the ideal ground for anti-science groups to the point of influencing government policy or being spruiked by political leaders [5]. Anti-vaxxer Facebook posts created doubt about COVID-19-vaccines long before vaccines existed, based on mistrust in the pharmaceutical industry, misinformation and conspiracy theories [6]. In tourism, misinformation became a serious issue for travellers, such as changes in risk perception or questioning public health measures, and residents, including the rise of racial discrimination [7, 8].

The traveller, who is potentially spreading the virus or being exposed to it, is part of the tourism industry. How did tourism respond to the emerging medical crisis? For a for-profit industry (as for stand-alone travel clinics), a downturn in travel is devastating. All global regions experienced a decrease in international arrivals, e.g., Europe and Africa by 85%, Asia and Pacific by 96% [9]. In the US alone, the pandemic led to $645 billion in cumulative losses for the travel economy through March 2021 [10], costing the US economy 41.1 trillion in economic output [11]. Forecasts and potential recovery strategies responded quickly [12–14]. However, to plan for a post-pandemic future, the tourism and hospitality sector had to look not only at balance sheets but focus more than ever on the heart of the industry, the travelling public, employees and residents, to understand their response, concerns and perceptions regarding current and future travel decisions. Many questions arise, for example, how tourism cooperates with health authorities and receives, responds and implements health directives, how the accommodation and food industry executes instructions, how the industry trains staff to be COVID-19-safe, how it looks after expats and local employees, and how these measures are communicated to instil trust in all involved. Furthermore, and directly important for travel medicine, what are travellers’ perceptions of the pandemic? How has travellers’ interest and confidence in travel been affected by constantly and rapidly changing, often perplexing, directives that were different for the same situation in different countries?

Medicine published on COVID-19 right away – and so did tourism academia. While travel medicine uses the combined evidence of several medical specialties to care for the health and wellbeing of travellers, tourism is much better in understanding them. Therefore, it is useful to take advantage of this knowledge to inform travel medicine and travel health clinicians’ work now and in the post-pandemic future. This article presents and discusses tourism research conducted over the first year of COVID-19 as it relates to people involved in the industry: travellers, employees and residents at destinations, the very population that is travel medicine’s focus of care.

## Method

At the time of the literature search (July 2021), the *Scimago Journal & Country Rank* site listed 123 academic tourism and hospitality journals. Starting with the top-ranked journals (from IP 10.982) and descending the list, ‘COVID’ was entered into English-language journals’ search engine. The search covered all papers published in 2020 with a focus on people. Later advance publications were added if data were collected in 2020. Excluded were papers on industry economics, e.g., forecasts, business, and management. This article starts with a general assessment of the targeted literature before addressing individual themes.

## Tourism literature’s focus on people in COVID-19 times

It is fascinating to witness the start of an entirely new thread of literature triggered by a novel topic of concern (see AIDS-literature in the 1980s). As soon as SARS-CoV-2 used travel to spread, the tourism industry responded to a threat that could devastate an entire industry. Rapidly designed research started as early as February 2020. Most papers present original research utilising social science or econometric methods and are highly complex, testing often more than ten hypotheses with the subsequent complex statistical analyses and presentation. Using mainly online surveys due to restrictions, the samples are large and comprehensive, resulting typically in an intricate network of causal relationships between variables. This much detail may be of no direct use to clinicians, but the overall trends give meaningful insights and inform travel health advice. Apart from references to the WHO, there are few cited medical sources; no paper was co-authored by health and tourism practitioners or scholars. Many studies originate in the Far East or in countries with high COVID-19 burden. Due to the delay between acceptance and publication, some statements are now outdated. Authors at the time could not foresee the duration of the pandemic, and one needs to think back to the early months of bewilderment and uncertainty when vaccination and the many attempts ‘to return to normality’ were unknown. As all authors had the same starting line, publications started independently without cross-referencing until later in the year. From 1 May 2020, the author guidelines of the high-profile *Journal of Travel Research* stipulated that submissions ‘must not ignore the effect of COVID-19’ [15]. An early warning to expect a COVID-19 research paper ‘tsunami’ prompted the call for a system-based research approach [16]. Lacking such a structure, papers presented here are organised into thematic clusters starting with risk perception and travel intentions including mass-gatherings, the use of technology to minimise infection, the impact of the pandemic on hospitality employees, residents’ reaction to travellers, hostility and discrimination, and a look into the future of tourism. Research recommendations for travel medicine, as they emerge from the tourism literature, conclude this discussion.

## The traveller – To travel or not to travel

Without tourists, there is no tourism. The rapidly evolving existential threat to the industry within the context of global bewilderment triggered studies focusing strongly on (potential) travellers. Researchers’ differing academic backgrounds guided the choice of research questions, resulting in a wide range of topics. These can be categorised into aspects out of people’s control and those where travellers play an active role.

Being subject to outsider-control and disallowed the freedom of movement one is accustomed to, impacts mental wellbeing [17], in line with the exceptionally diverse psychology of pandemics [18]. Denied mobility, the ‘lockdown captivity phenomenon’[19] and ‘travel craving’[20] have been studied in Italy and Hungary in May/June, while an analysis of *National Geographic* promoted Instagram posts in April demonstrated a marked change in expression of personal experience and skills, social facets and in lifestyle [21]. In the pre-travel stage, in April, sentiment analysis on over 600 Italian online posts showed customer concern with airline cancellation and compensation, but also the much-unexpected result that a rise in COVID-19-deaths, not cases, increased empathy with struggling airlines [22]. In February, three US-studies demonstrated that the threat of infection decreased the willingness to accept price inequalities [23].

Other aspects can be controlled by travellers: the avoidance of crowding [24], or a willingness to pay for enhanced safety measures [25–28]. Most importantly, adherence to PNPIs lies very much with the individual. End of April, of over 400 Kosovar, 90% planned to travel to Albania that summer. At the time, 15% did not follow strict health ministry directives, 25% sometimes to never socially distanced, while 28% sometimes to never wore masks appropriately around others [29]. In the US, men were more likely to refuse masks for international travel [30]. In China, more women adhered to pro-social behaviour [31]. A far greater area of research covers people’s risk perception and subsequent intentions to travel during and after the pandemic.

## Risk perception and travel intentions

The notion of the ‘crisis-resistant tourist’ who ‘travels despite’ or ‘not cancels because of’ crises, but does not risk-shift, i.e., take out travel insurance more than others [32] may help appreciate the following results. Because of the economic impact of reduced/cancelled travel, border closures and lockdowns, the industry is keen to understand potential travellers to be able to develop strategies to get people travelling again. First, it is especially important to appreciate people’s perception of risk during a pandemic.

### Risk perception

Travel medicine is interested in risk perception (274 hits in *Journal of Travel Medicine* and 134 hits in *Travel Medicine and Infectious Disease*, 22 Dec 2021) because it influences decisions to visit a travel clinic, destination and behaviour choices, and subsequent adherence (or not) to health advice. For tourism, risk perception influences business decisions to secure profits. In contrast to real risk, perceived health risks are based on cognitive, affective, individual and contextual components and, therefore, subjective [33], influenced, for example, by the media [1, 2, 34]. In the first four months of the pandemic, media coverage influenced risk perception in Korea more than case numbers [35]. In two secondary data analyses from Hong Kong combined with four original US-surveys (*n* = 744), the perceived threat of infection increased the tendency to avoid extreme travel options [36]. A South African survey (May/June) of 323 potential tourists from Africa, Europe and Asia assessed psychographic factors: dogmatic, sceptical and apprehensive, depending on risk perception and level of caution [37]. Important for travel medicine, dogmatic tourists may not follow risk mitigation measures. Over 1000 Indian travellers perceived risk differently depending on their fear of infection. Women, married and older travellers saw COVID-19 as more severe and adopted PNPIs more readily. While education made no difference in risk perception, higher education levels increased PNPIs usage. People on lower incomes and travellers for work and education were less willing to implement such measures [38]. Surveys in Germany, Austria and Switzerland (*n* = 1370) before and immediately after the WHO-proclamation demonstrated relative low concern about COVID-19 before but strong increase in risk perception after, viewing as irresponsible business trips and travel to destinations with cases. Contact with tourists in one’s hometown were to be avoided [39]. Mothers avoided business trips to protect their family from potential infection [40].

Consumer distrust in hotel hygiene standards existed before, based on the assumption that service providers act negligently and incompetently [41]. Almost 99,000 Chinese hotel reviews of all 185 five-star hotels in Shanghai demonstrated a shift in consumer preferences beyond hygiene expectations. Breakfast, location and surroundings lost importance while in-hotel, in-room experience, service, cleanliness and front desk gained importance due to the ‘cocooning’, i.e. staying in hotel rooms for one’s own safety [42]. Staying in ‘love hotels’ in Ho Chi Minh City rather than tourist hotels appeared less risky, possibly because of less crowding and low tourist contact [43].

End of 2020, US restaurant and hotel customers (*n* = 809) were reluctant to eat in sit-down restaurants, wanted visible evidence of sanitising efforts and accepted technology and robots [25]. A comprehensive exploration of tourists’ risk perception of COVID-19 proposed a conceptual model to interpret and explain travellers’ behaviour patterns. The section on risk included the obvious health and psychological risks as well as social risks (disapproval of one’s travel plans), performance risk (not receiving expected service), image risk (stigma of a location), and time risk (time-related costs, quarantine) [44].

For tourism and travel medicine, it may be important to consider shifting the public’s concentration on risk avoidance to risk management [45]. Normally, travel insurance provides some reassurance in case of misfortune. However, most insurer policies exclude pandemics and known events, and travellers were unable to purchase cover when they most needed it, leading to a ‘reverse moral hazard effect’, i.e., a reluctance for future travel. Yet, insurers need to exclude catastrophic events to remain solvent [46]. Willingness to pay (WTP) for perceived or actual better service/goods is a well-known tourism concept, which also emerged during the pandemic. WTP related to the expectation of particularly stringent health measures on transport, in restaurants and accommodation, for example, in Italy where, however, WTP was lower in regions with COVID-19 [28]. Because high adherence to hygiene measures was expected, no WTP was evidenced in Spain, especially when there was a strong intention to travel [47]. To manage risk, apart from WTP for superior crisis management, some type of travel allows crowd avoidance, for example, camping [27] or a ‘safe’ destination, e.g., geological sites in Oman [48]. To enable the public assessing a location’s safety from epidemics as part of travel preparations, a ‘country-level index of epidemiological susceptibility risk’ was proposed built on health infrastructure, demographics, environmental safety infrastructure, economic activity, communications infrastructure and governance institutions [49]. The onus would be on tourists to assess if the chosen destination can deal with potential risks adequately – a formidable task. It is unclear who was to compile this index, especially in poor countries. Regardless how this risk is perceived, what counts for travel medicine and tourism is the public’s actual plan to travel.

### Travel intentions

Intentions differ from desire as they are perceived as more realistically ‘do-able’ within a firm timeframe [50]. At the time of data collections, as everybody else, neither researchers nor study participants knew how long this pandemic would last or if there was a clear end. Nobody knew of the varying individual and global policies and travel bans or about potential vaccines. Therefore, people’s travel intentions could not always be classified clearly as during, post-pandemic or loosely ‘sometime later’, nuances usually lost in quantitative data. Interviews with potential travellers and tourism professionals in Western Australia formed the basis for a motivator-demotivator approach to travel during COVID-19. Motivators were needs for mental wellbeing and social connectedness including personal growth and relaxation. Demotivators consisted of health and safety risks including the level of perceived competence of authorities to handle the crisis [51].

In Greece, travel was unlikely not because of COVID-19 but lack of funds [52]. In Italy – as in Egypt [53] – trust in responsible provision of safety protocols influenced travel plans [28]. In May, among 1144 18–90-year-old Italians, age influenced vacation preferences [54]. In the same month, students and workers in Macau reduced their travel intentions but felt safe due to strict policies; however, tourists were urged to stay away [55]. In contrast, in India, intended travel did not necessarily mean adoption of PNPIs [38]. Unexpectedly, in June, Spanish data suggested that living in an area with worse case numbers and having personal experience with the disease, increased plans to travel this very summer, especially in men and those very concerned about the pandemic. Adhering to health rules, such travel may be mentally beneficial [56]. Some intended travel leads to the accumulation of large numbers of people, for example, travelling on cruise liners or gathering for religious or cultural festivals.

## Mass-gatherings

The close distance between people and their mingling at large gatherings provides the perfect scenario for ‘super-spreader’ events.

### Cruises

One such example are ocean liners where large numbers of passengers are inescapably confined by the perimeter of the vessel. The unfortunate outcome revealed itself in the dramatic events on cruise ships early in the pandemic. On 1 February, a passenger leaving the *Diamond Princess* earlier in Hong Kong tested positive. The ship arrived in Japanese waters on 3 February, and 3711 passengers and crew were quarantined [57]. On 19 March, 2650 passengers disembarked the *Ruby Princess* in Sydney before COVID-19 test results were known, to avoid missing connecting domestic and international flights. This mishandling became the single largest source of Australia-wide infections [58] resulting in over 900 cases around the country and 28 deaths [59]. Especially frightening for the public were the rapid deaths, the first on 24 March. On 15 March, four days after the WHO declared a pandemic, and while companies cancelled cruises and ships at sea were denied access to ports, the Australian luxury expedition ship *Greg Mortimer* left Argentina for Antarctica with 217 people on company advice that no virus was on board. Day 8 recorded the first fever. The original itinerary abandoned, and Argentinian ports closed, Uruguay allowed docking offshore. Eight passengers and crew were evacuated, including one ship physician. Of all 217 on board, 128 (59%) tested positive [60]. One Filipino crewmember died [61]. What polished written reports cannot convey is captured brilliantly in the 2-part documentary *Deadly Trip of a Lifetime* [62, 63]. Staff are often forgotten when the focus is on travellers. After passengers disembarked, the crew of many ships were stuck at sea, often confined to their small windowless bunks instead of being moved to the then vacated passenger cabins, away from their families, with often limited communication and, in some cases, exposed to irresponsible company pressures [64–66]. Staff’s mental distress led to a number of alleged suicides on-board [64].

By the end of March, many ships were still wandering the high seas unable to find a port to dock. The cruise industry came to an abrupt halt with massive economic losses. Trust in a company’s crisis management was essential for lower-income US-travellers who were willing to cruise again with a steep discount [67]. In contrast to new customers, influenced by other consumers’ negative experiences, repeat customers were guided by their own previous experiences [67]. This interesting concept could be explored in travel medicine research on risk perception of new vs repeat travellers in general.

Trust in government/public health agencies and cruise companies played an important role in risk-reducing behaviour and future cruise intentions of 504 Australians. To regain trust, the perception of competence, consistency, consideration (in the best interest of public) and conviviality (good will toward the information provider based on trust) will need to be restored [68]. Almost 55,000 tweets (1 Feb – 18 June) reflected the global public sentiment toward cruising, mirroring the evolving events during the early pandemic. A growing interest in river cruising showed attempts to gain distance from the masses [69]. Legal questions regarding humanitarian obligations to assist cruise passengers in need vs a country’s obligation to safeguard its population [70] also involve health and medicine.

### Religious tourism

Religious travel spans from crusades, historic pilgrimages, and missionary travel to today’s faith-based conventions or retreats. Modern day international examples are Hajj and Umrah, the Shia pilgrimage to Iran and Iraq, Kumbh Mela in India, Easter at the Vatican, or Christmas in Bethlehem, and smaller local festivities. A pandemic requires sudden decision-making of health authorities at the faith-based destination, e.g., the Ministry of Hajj and Umrah [71] and in countries of pilgrims’ origins [72]. Cancelled in 2020, in 2021 only 60,000 vaccinated pilgrims were admitted to the Hajj. Not only is overcrowding of concern, but the touching/kissing of objects such as walls of shrines [73] or statues of saints. There is a clear concern for the economic effect on religious destinations [73, 74], and the impact of COVID-19-measures on the faithfuls’ ability to follow religious obligations.

Appreciating the role faith plays in a crisis, WHO published in April 2020 practical recommendations for religious leaders and faith-based communities, asking to share clear, evidence-based steps to reduce fear, provide reassurance and promote health-saving practices [75]. The detailed guidelines focus on gatherings, safe burial practices and leaders’ role in COVID-19 education. The recruitment of religious leaders was crucial with the introduction of vaccines. While Pope Francis saw vaccinations as a moral obligation [76], others warned of vaccines causing homosexual tendencies, inserting microchips, or being produced from cow’s blood (to harm Hindus) or slaughtered foetuses [77]. In the Serbian Orthodox Church, Holy Communion during Easter is of highest importance as medicine for soul and body. The church’s appeal to observe health directives was met with strong resistance and many requests to lift the travel ban during Easter. The ban represented not only ‘physical’ social distancing, but social (and religious) distancing in its true sense [78].

An Indonesian study compared pre-Eid travel intentions in February 2020 and actual travel (despite a travel ban) after festivities in May. Lack of travellers’ personal agency, e.g., perceived obligation to religion and family, promoted risky behaviour and ‘wished away’ potential health risks [79]. In India, before the Delta variant, people were willing to continue travel post-COVID-19 to religious sites provided reliable health and safety measures were in place during travel and stay [80]. An often-overlooked travel situation is being stuck overseas due to unforeseen events. A study with Pakistani pilgrims to Iran, unable to return home, explores the topic of travel burnout [81]. Where normally spirituality is a source of well-being, pilgrims were confronted with unexpected out-of-their-control situations of border closures, delays, need for food and shelter on top of the fear of becoming infected. Pilgrims showed low self-efficacy (existential fear, xenophobic response on return, restricted mobility), travel exhaustion (stress, new protocols, friction among the group, homesickness) and emotional maladaptation. Coping strategies included faith, better future travel planning, and reliance on friends and family. Coping with being trapped unexpectedly during travel is much under-researched and fits easily the travel medicine research portfolio.

## Technology meets health directives

The understanding that close human contact, an important part of travel, increases the spread of infection, prompted tourism to find ways to provide safe travel experiences, using robots and virtual travel. Artificial intelligence devices have been employed in tourism previously and consumers’ attitudes towards them studied eagerly [82]. Now they are an important attempt to minimise person-to-person contact with the bonus of frequent sanitising.

During COVID-19, anthropomorphic robots, robotic vehicles and other autonomous devices were used in hospitals, communities, airports, recreation areas, and hotels and restaurants [83]. There are challenges, as in the unfortunate *Henn na Hotel* in Nagasaki [84], but also job losses, privacy and data security, misuse by governments [83], and a robotic barman unable to listen to personal problems. However, in pandemic times, the acceptance of robots may be greater [85]. Just before COVID-19, over 500 TripAdvisor reviews (2013–2019) of three robotic hotels in the US and Japan were positive, though the sample may be biased towards technology-fans who enjoy robots as added preference. In a pandemic, robots could assist those who want to travel [86]. As physical distancing reduces the risk of infection, 1062 US and Chinese customers’ risk perception when interacting with hospitality staff influenced their acceptance of service robots [87]. Tourists from 18 countries preferred anthropomorphic robots to all other types, but robots should not replace the innate anthropocentric nature of travel. The increased use of robots during and after COVID-19 may change acceptance as a means to avoid infection [88].

Travel bans, lockdowns and social distancing favoured the increase of webcam-travel and virtual tours – free or purchased. Though of differing quality, technology brings attractions to the ‘traveller’s’ home. University students and staff (*n* = 401) in Oman and Germany found virtual travel beneficial for the disabled and those less affluent, and during lockdown or crises. Not replacing real travel, it could entice people to visit the actual site after the pandemic [89]. Locals, of course, gain little from virtual tourism. US citizens suggested that perceived high COVID-19 threat severity, response efficacy and self-efficacy raised social distancing behaviour which increased the likelihood of using virtual tours, while those with perceived low threat severity continued to travel in person [90]. Feelings of freedom, nostalgia and connection triggered by webcam-travel were associated with happy memories made before lockdown, and so uplifted people’s mood [91].

## The impact of COVID-19 on hospitality employees

While a pandemic can cripple an industry economically, an industry only exists on the shoulders of employees who are not only personally at risk of infection but experience a dramatic change in demands on them. Tourism workers suddenly had to clean, serve, communicate, distance, and implement bespoke instructions without a health background, much like the general public who was supposed to follow rules without understanding the link between the required activities and viral behaviour. The first studies into the impact of COVID-19 on tourism workers focussed on hotel and hospitality employees. The comments of 36,793 employees on the US-site Reddit, posted 3 January to 19 April, displayed real-time perceptions. Up to April, anxiety led all other negative emotions, when anger joined other factors, such as employment and racism [92]. In Turkey, 151 staff from two 5-star hotels responded in June to the risk of infection with increased mental health problems, absenteeism and low life satisfaction, the latter somewhat balanced by being married with children. Companies should, therefore, demonstrate a level of care by offering stress management programs (resilience, alcohol, finances), affordable groceries and medical care [93]. Unemployment, pandemic-induced panic and lack of social support caused distress in US tourism employees (*n* = 1231), especially in women and young employees [94]. US immigrant hospitality workers, disproportionally represented in hotel and food services, on low wages and poor working conditions, were even more affected considering their ineligibility for COVID-19-aid despite paying taxes [95]. A company’s response to COVID-19 influences employees’ perceptions on risk. In Vietnam, a surprising result was obtained from almost 400 employees in that satisfaction with the organisation not only helped raise job performance but strengthened the positive effect of a perceived health risk on job performance; full trust in organisations allowed concentration on the job [96]. This might indicate the importance of an employer when lacking national relief polices; it could also mean that desirable responses were collected.

Socially responsible workforce management influences employee anxiety. Over 400 Chinese tourism workers (almost half from Wuhan) indicated in February the importance of trust in the organisation to overcome fears, especially of unemployment, and poor mental health [97]. Similar results arose from 1594 employees from 23 Chinese hotels. Close person-to-person contact makes hotel-employment a high-risk occupation. Using the constructs: safety coaching, control, motivation, care, compliance, participation, adaptation, perceived susceptibility, perceived severity and belief restoration, employee perception of a hotel’s socially responsible initiatives promoted compliance with specific directives and citizenship behaviour. Hotels should assist employees in managing perceived risk by providing objective up-to-date information, assisting in dealing with negative emotions, providing stability, developing emergency response plans and support belief restoration [98, 99]. While COVID-19 highlighted the immediate effect on tourism workers, the question arose if this is, indeed, a different situation ‘from the precarious lives they normally lead or just a (loud) amplification of the “normal”’ [100,p. 2813]. The authors propose that hospitality work in a pandemic is a magnification of misery, not something new, and highlight the problem at three levels. At the top level (macro), governmental, international agency and global policies ensure a framework of low wages, poor working conditions, and insufficient social security, e.g., ‘flags of convenience’ with uncontrolled exploitation of cruise ship workers. At the meso level, organisations control through outsourcing, ‘business hibernation’ and furloughing. In a pandemic, this leaves the employee at the micro level even more vulnerable to crises, especially young, women, immigrants, and international student workers [100].

## Residents’ reaction to travellers during COVID-19

An important part of the tourism experience is the interaction with local people who, in general, and even if only for economic reasons, welcome visitors. Does this welcome change with visitors potentially bringing disease? In February, comparing the perception of social cost (shortage of necessities, travel restrictions, pressure on hospital beds) of a combined 3364 residents in Hong Kong, Wuhan and Guangzhou by using two hypothetical scenarios, confidence in authorities was easily lost when policies were compiled hastily. Positive framing of messages and ‘mental accounting’ of pros and cons, based on evidence, are important to ensure trust in directives [101]. In the same cities, in February/March, 1627 residents were most concerned about the risk of cross-infection due to tourism activities and, especially younger people, showed a WTP for risk reduction and appropriate action [26]. From March, and for a year, a qualitative study monitored the impact of COVID-19 on tourism in Bali. Already a mass tourism destination producing 55% of GDP, Bali’s original plan was to increase international arrivals to 20 million in 2020. While the Balinese people followed health directives, initially without any official advice for the tourism industry and with rising case numbers and deaths, those dependent on tourism had grave fears for their economic survival. On the other hand, those without links to the industry saw the break in arrivals as a welcome pause in ‘over-tourism’ and pointed to the need for more respectful, sustainable approaches. For them, COVID-19 was a wakeup call from God to the Balinese regarding the unsavoury sides of tourism. The official line, however, appears to support a return to mass tourism to make up for the losses [102]. In May/June, 634 residents on the Korean Jeju Island, which experienced an increase in domestic tourism, indicated that the perceived risk of being infected by visitors influenced their level of welcoming emotions. Residents cannot identify infected tourists. In contrast to tourists who can avoid hotspots, residents cannot leave [103]. The dilemma between supporting the economy and risking infection emerged from a Japanese survey. The ‘Go to Travel’ campaign, providing discounts and vouchers to increase domestic travel, was unwelcome by many. Even if residents followed all health directives, they could not escape tourists [104].

The vulnerability of indigenous destination communities has been of concern. They suffer equally a loss of business, but being often in remote or isolated settings and further away from suitable health care, infections would be disastrous. In Australia, most indigenous communities were off-limits to individual and organised tourists. Canada [105], New Zealand [106] and Brazil [107] voiced similar concerns with a shift to more emphasis on social and environmental wellbeing and respect rather than the insistence on the ‘right to travel’[105].

## COVID-19 and travel: hostility, discrimination, racism

Fear of infection also shows in discriminatory reactions of residents to visitors. Press reports emerged very early on from India of international tourists being directed to leave accommodation and country, refused food or met with severe hostility [108, 109]. Even more pronounced were aggressive reactions around the world towards not only travellers of Asian appearance but also residents in non-Asian countries [110–112]. Chinese international students in the US found that their mask wearing indicated illness and put them at even greater risk of racial abuse [113]. In February/March, 26 tourists to India reported a sense of mistrust towards tourists, subsequent negative emotions towards India and a lack of willingness to interact with locals due to the perceived rejection, but also an observed lack of implementation of health directives [114]. A similar link between unwelcoming resident behaviour and destination perception emerged in Hong Kong [115]. In February, 203 US citizens indicated that residents who experienced everyday discrimination themselves based on some social attributes, were more likely to support hostile responses against tourists, especially Mainland Chinese [116]. A study on host–guest relations in Singapore mid-2019 offered a chance to compare such views with those in April 2020 (combined *n* = 468). Before COVID-19, Mainland Chinese were tolerated for their spending power despite being stereotyped unfavourably. Perceived risk of infection and expected restrained spending may lead to increased intolerance towards these visitors [117]. Much blame for this discrimination lies with the media [7, 8].

Tourism has studied xenophobia before. The xenophobic tourist anticipates and/or experiences unpleasant emotions related to the encounter with locals at foreign destinations. For example, the more xenophobic a traveller, the higher the uptake of travel vaccination, insurance, group travel and booking through an agency, and the lower the interest in local food. Men were more xenophobic; education or age made no significant difference [118]. This deep-seated unease extends to purchasing behaviour in general, e.g., buying local products, but also choosing familiar airlines and hotels when travelling to a destination similar to home [119]. COVID-19 added the unpleasant perception of crowding [120].

Tourists’ fear of the ‘other’ (host) originates from the same ancestral disease-avoidance mechanism as the fear of residents of the ‘other’ (visitor). In ancestral social groups (in-group), people learned about the potential ill effect from contact with people from other social groups (out-group) and developed adaptive behaviours. Based on cues of ‘strangeness’, i.e., an otherness to one’s own ‘normality’, out-group people were avoided not only for cues, such as their physiognomy, food and hygiene practices, but the perception of vulnerability to potential disease. Negative attitudes including disgust then led to the culturally evolved behaviour of keeping a distance [121]. Furthermore, staying within one’s own group poses less of a risk of disease transmission as well as ensures the likelihood of being cared for and supported in need [122]. This ‘behavioural immune system’, the avoidance of contact and sticking to the in-group, is easier to implement [123]. After all, pathogens are invisible; therefore, other cues need to be employed. This leads to in-group conformity and out-group exclusion [124]. However, this exclusion also applies to in-group members who had the misfortune of being caught out at an out-group location, such as Balinese cruise ship workers returning home [102] or Pakistani pilgrims returning from Iran [81]. Having limited or no control over COVID-19-events, people’s own locus of control may also attribute blame, for example, on destinations [125] or on marginalised people, such as refugees and asylum seekers [126], and perhaps, in the future, the unvaccinated. Evolutionary motives are the ultimate explanation of discrimination during COVID-19, but this does not condone the widespread hostility experienced by travellers and residents alike. Media misinformation and conspiracy promoters have much to answer for, although health and medicine have not excelled in improving general health literacy on which to base appropriate health information in the event of a pandemic.

## Future directions in tourism

For decades, scholars have warned of negative outcomes through relentless growth in tourism. As late as 2019, these warnings demanded a ‘de-growing’ and reprioritising, while proposing wide-ranging strategies for change [127], strategies widely ignored by corporate giants. Ironically, just one year later, COVID-19 showed precisely not only the trouble tourism had created for itself, but also how it contributed to the spread of the virus. To salvage some profits, like everybody else, business owners and executive boards had to make decisions based on knowledge of the virus, constantly changing government and public health directives and their different interpretations in different countries, personal opinions of health professionals, poorly constructed messages to the public, often questionable media involvement and crass conspiracy theories. The questions arise how the pandemic has shaped our desire to travel, and what tourism will look like after the crisis. Two aspects may support a change in direction, long asked for by tourism scholars and residents at destinations.

First, lengthy lockdowns and restrictions have modified many people’s worldviews, lifestyles and previous behaviours. Mindfulness, ‘slowing-down’, a measured approach to consumption and a focus on ‘what is really important’ gained prominence, at least for those who can afford such luxury. This view may now extend to many more travellers beyond those who travelled mindfully before. Second, media reports of wildlife moving into seemingly abandoned suburbs, cleaner water in rivers and oceans, better air quality, less waste (apart from an unprecedented increase in medical waste [55]), and peace and quiet showed an almost forgotten picture of a different world. Considering tourism’s involvement in and suffering from COVID-19, how the industry will progress from here is important for travel medicine as it may influence travellers’ different care requirements depending on changes in destinations or holiday activities. There are two opposing schools of thought: either return to growth and mass tourism or take advantage of the opportunity to reset.

The first view is that tourism must recuperate the enormous losses and get ‘back to normal’ as soon as possible, trusting that people have short memories (shortly after the *Ruby Princess* debacle, long waiting lists for the next possible cruise filled quickly). Opening borders, spare funds, boredom, and fear of missing out may lead to ‘revenge travel’ or ‘catch-up travel’ [128] without considering impacts or consequences. The economic benefits of tourism, driven by the World Tourism Organisation and supported by government interventions, may again be the driving force behind the ‘business-as-usual’ return to pre-COVID-19 business behaviour, a possibility that sparked a fiery debate between the two tourism camps [129]. This dilemma is evident in Bali, where residents who depend on tourism desperately want it back while others relish having the island to themselves. Government intentions seem to favour a return to growth-tourism [102]. Similar concerns apply to Nepal, which had declared 2020 the ‘Visit Nepal Year’, with a potential return to excessive over-tourism that prevailed before the pandemic [130].

The second view, recognising that mass-tourism is not resilient and inert in responding to sudden changes, suggests treating the pandemic as a chance to transform global tourism away from unsustainable and destructive growth towards mindful and equitable forms that prioritise quality over quantity [131, 132]. Suggestions are a preference for slow nature-focused tourism [133] and its mental health benefits [134], avoidance of mass-cruises [135] and a greater consideration for host communities [136]. In April 2020, *Tourism Geographies* devoted a highly recommended special issue to the discussion of how COVID-19-events can contribute to a ‘substantial, meaningful and positive transformation of the planet in general and tourism specifically’ [137,p. 455] where growth is in well-being, not profit. This goes far beyond the call for responsible tourism, i.e., the call for having less damaging impacts, and requires a radical transformation away from systematic inequalities [138] towards a balanced, resilient and just post-pandemic tourism [139, 140]. Pleasingly, small operators may turn out more resilient due to their potential flexibility within a specific local community than unwieldy multinationals [141].

Compared to previous pandemics and large-scale disease outbreaks over the last 100 years, COVID-19 will be the costliest, at least in economic terms. While some locations may opt for a mindful change, it is highly likely that the focus remains on growth, which may prove even more unsustainable than before [142].

## Recommendations for research

Looking at other disciplines’ research topics and methods can unearth useful ideas adaptable by travel medicine for better travel health care and understanding of travellers’ motives, attitudes and behaviour [143]. The criticised lack of a structural research agenda at the beginning of the pandemic [144] and the subsequent flurry of diverse topics and approaches nevertheless provides travel health practitioners with a vast range of frameworks, topics and methods useful in novel travel medicine research. Theories, such as the Protection Motivation Theory, Theory of Planned Behaviour, Risk Aversion Theory, Attribution Theory (Locus of Control), Cognitive Appraisal Theory, Theory of Reasoned Action, Motivations Reasoning Perspective, and many more are useful to study travel health behaviour, risk perception, coping strategies and so on, thereby elevating the usual KAP (Knowledge, Attitudes and Practices) studies to a more robust level. Equally, several tested tools could be explored and modified to suit travel medicine concerns, such as the Tourist Worry Scale [145], Tourism Fatigue Scale [146], Travel Safety Attitude Scale [147], Pandemic Anxiety Attitude Scale [148], Tourist Xenophobia Scale [118], or Sentiment Analysis [69] for text-mining of social media data.

This article has covered a wide range of topics, all of which could be examined from a travel medicine perspective and in multidisciplinary teams, the latter a particularly valuable way to develop fresh research questions [149]. The impact of infectious disease on travellers’ psychological state [23], distrust in service providers [41] including travel health providers, perceptions of inconsistent/conflicting medical advice, vaccine acceptance influenced by religious leaders or ‘anti-vaxxers’, or the acceptance of travel health advice during a pandemic are only some examples. Assaf et al. suggested 17 topics for future research for consumer/traveller behaviour alone [150]. Many harbour health aspects. The effects of sensational media coverage regarding travel medicine concerns are little understood. Discrimination and racism may influence certain health behaviours abroad, e.g., choosing familiar food from questionable hotel kitchens over freshly prepared ‘foreign’ local food. For more detailed insight, the times of data collection for the presented studies could be linked to the respective country’s case numbers, health directives, government policies, travel restrictions and lockdowns at that time, for example, matching the medical response in Vietnam [151] to a study on employees in Vietnamese hotels [96]. Travel medicine research usually focuses on travellers’ wellbeing. So far, travel health professionals themselves, especially during a pandemic, have been of limited interest to researchers.

## Limitations

This article only utilised English-language academic tourism journals, potentially missing important findings. Journals of other specialties, such as aviation, transport, food and catering, were not consulted. No doubt, there were many manuscripts still in the peer-review or revision phase. With evolving knowledge of the virus’ behaviour and subsequent policy responses, there may be a shift to an entirely different focus of concern in later studies.

## Conclusion

Even before the pandemic was announced, tourism scholars recognised the existential threat to the industry, reacted quickly and commenced research depending on their respective area of expertise. Although these early studies were, naturally, uncoordinated, many focused on the lifeblood of tourism: travellers, workers and residents, the very core of travel medicine. Parallel interests emerged. Risk perception and travel intentions are examined here from the industry’s perspective. Health directives advise strongly against mass-gatherings, yet people insist on getting on cruise ships as soon as possible or wish to follow religious or cultural obligations. On the other hand, technology in the shape of virtual travel or robotic devices keeps people at safe distance and so minimises person-to-person contact. The impact of COVID-19 on tourism workers and residents at destinations including the arising hostility and discrimination, are firmly based in a health context. If and how tourism learns from the current business model’s vulnerability will affect travel health practitioners’ work.

While the results are tourism results, they allow a better insight into people than travel medicine research typically can, with implications for travel health practitioners. If travellers are reluctant to travel for a long while, travel health clinics lose revenue and practitioners may lose recency of practice. People who will travel regardless may present to clinicians different sets of issues, require a modified approach to travel health advice, ask different questions, e.g., ‘do robots really protect me?’, or state their distrust in (health) authorities and so challenge practitioners to provide evidence so that travellers can make sensible informed decisions.

Travel medicine and the tourism industry are tightly connected via the traveller, yet there is still little cooperation, collaboration and acknowledgment of the other. This connection should be exploited more for the benefit of travel health and medicine and, ultimately, for the traveller. The first 6–12 months of the pandemic seem now a long time ago due to vaccination, anti-viral treatment and adoption of a ‘new normal’, with the realisation that COVID-19 will not disappear in a hurry. It is prudent to remember those first months and the ‘hits and misses’ in medicine and tourism. Presumably, the next pandemic is aided by travel again – and may be just around the corner.

## Data Availability

Not applicable.
